# Association of Novel Androgen Receptor Axis-Targeted Therapies With Diarrhea in Patients With Prostate Cancer: A Bayesian Network Analysis

**DOI:** 10.3389/fmed.2021.800823

**Published:** 2022-01-24

**Authors:** Xingyu Xiong, Hang Xu, Sheng Wang, Xinyang Liao, Xianyanling Yi, Kun Jin, Haoran Lei, Shengjiang Bai, Shi Qiu, Lu Yang

**Affiliations:** ^1^Department of Urology, Center of Biomedical Big Data and National Clinical Research Center for Geriatrics, West China Hospital of Sichuan University, Institute of Urology, Chengdu, China; ^2^Center of Biomedical Big Data, West China Hospital, Sichuan University, Chengdu, China

**Keywords:** novel androgen receptor axis-targeted therapies, diarrhea, constipation, prostate cancer, network meta-analysis

## Abstract

**Objective:**

To perform a systematic review and network meta-analysis to characterize the effect of novel androgen receptor axis-target (ARAT) agents on diarrhea and constipation.

**Methods:**

We searched the Pubmed, Web of Science, and ClinicalTrials.gov up to September 2021 for phase 3 randomized controlled trials (RCTs) of patients receiving novel ARAT agents for prostate cancer (CaP). A Cochrane risk-of-bias tool was used to assess trial quality. The primary outcomes were risk ratio (*RR*) of any-grade diarrhea and constipation for patients receiving ARAT treatment. *RR*s of competing treatments were evaluated by pairwise and Bayesian network meta-analysis.

**Results:**

In this study, 13 trials with 15,117 participants comparing 5 treatments (abiraterone, enzalutamide, apalutamide, darolutamide, and placebo) were identified. Use of novel ARAT agents was associated with a significant increased risk of any-grade diarrhea (*RR* = 1.30, 95% *CI* [1.16, 1.44]). As for subgroup analysis, abiraterone, enzalutamide, and apalutamide were all associated with significant increased risk of any-grade diarrhea (abiraterone: *RR* = 1.40, 95% *CI* [1.09, 1.81]; enzalutamide: *RR* = 1.17, 95% *CI* [1.02, 1.35]; apalutamide: RR = 1.35, 95% *CI* [1.03, 1.76]). Based on Bayesian modeling, abiraterone and enzalutamide showed the highest and lowest probability to rank first in terms of increasing risk of any-grade diarrhea. There were no significant differences of risk in any-grade constipation, grade 3 or greater diarrhea, and constipation between ARAT and control group.

**Conclusion:**

The present study indicates that the use of novel ARAT agents is associated with a significantly higher risk of diarrhea. Across the four agents, abiraterone may relate to the highest risk of diarrhea among patients with metastatic hormone sensitive prostate cancer (mHSPC) and castration-resistant prostate cancer (CRPC).

## Introduction

Prostate cancer (CaP) is the most common cancer in men, which accounts for 26% of diagnoses in the United States in 2021 (1). Androgen deprivation therapy (ADT) remains the mainstay of treatments in patients with advanced CaP (2). However, almost all patients invariably developed from hormone sensitive prostate cancer (HSPC) to castration-resistant prostate cancer (CRPC) (3). In recent years, several novel androgen receptor axis-targeted (ARAT) agents, such as abiraterone (Abi), enzalutamide (Enz), apalutamide (Apa), and darolutamide (Dar), were developed to further inhibit the AR signaling in patients with CRPC. Additionally, five large randomized controlled trials (RCTs) indicates that the addition of Abi, Enz, or Apa to ADT in men with metastatic HSPC (mHSPC) could significantly improve overall survival and progression-free survival compared with ADT alone (4–8).

Improved prognosis has created growing needs to address the unique health issues facing CaP survivors that result from CaP, its treatment, and related comorbid conditions. Previous studies have demonstrated that gastrointestinal (GI) complications, such as diarrhea and constipation, are one of persistent burdens for CaP survivors treated with ADT (9–11). Although the mechanisms associated with GI complications in CaP survivors are poorly clarified, available evidence indicate that a dysbiotic composition of GI microbiota may mediate GI complications in CaP survivors (12, 13). Furthermore, there is emerging evidence that circulating androgen levels and castration can affect the composition of GI microbiota (14–16). Recently, Sfanos et al. demonstrated that oral hormonal therapies, such as Abi and Enz, for CaP could alter the intestinal bacterial composition of fecal samples from rectal swabs (17). Furthermore, radiotherapy, chemotherapy, and immunotherapy have been demonstrated to induce dysbiosis that was associated with treatment toxicities, such as diarrhea (17, 18). It could be hypothesized that novel ARAT agents might further impact the function of GI and cause GI complications through altering composition of the GI microbiome. However, a paucity of research regarding this issue exists.

This study aimed to determine the effect of novel ARAT agents on GI complications utilizing the reconstructed clinical data derived from phase 3 RCTs to inform decision-making. As most of included trials only report part of GI complications, we focus on two of the most reported complications which are diarrhea and constipation. Additionally, diarrhea and constipation are both most concerned GI complications of cancer therapies.

## Methods

### Search Strategy and Selection Criteria

We conducted a systematic review and network meta-analysis followed the Preferred Reporting Items for Systematic Reviews and Meta-analyses (PRISMA) reporting guideline and its extension for network meta-analysis (19, 20). We searched the Pubmed, Web of Science, and ClinicalTrials.gov up to September 2021. The following searching terms were used: [“Prostate Cancer”] AND [“Abiraterone” OR “Enzalutamide” OR “Apalutamide” OR “Darolutamide”]. We performed the study eligibility using the population, intervention, comparator, outcome, and study (PICOS) approach: (P) studies focused on patients with a diagnosis of CaP; (I) treated with Abi, Enz, Apa, or Dar; (C) in which placebo was performed as a comparator; (O) reporting one or both of the following outcomes: diarrhea and constipation; (S) in phase 3, placebo-controlled, double-blind, and randomized trials.

### Study Selection and Data Extraction

**Figure 1:**
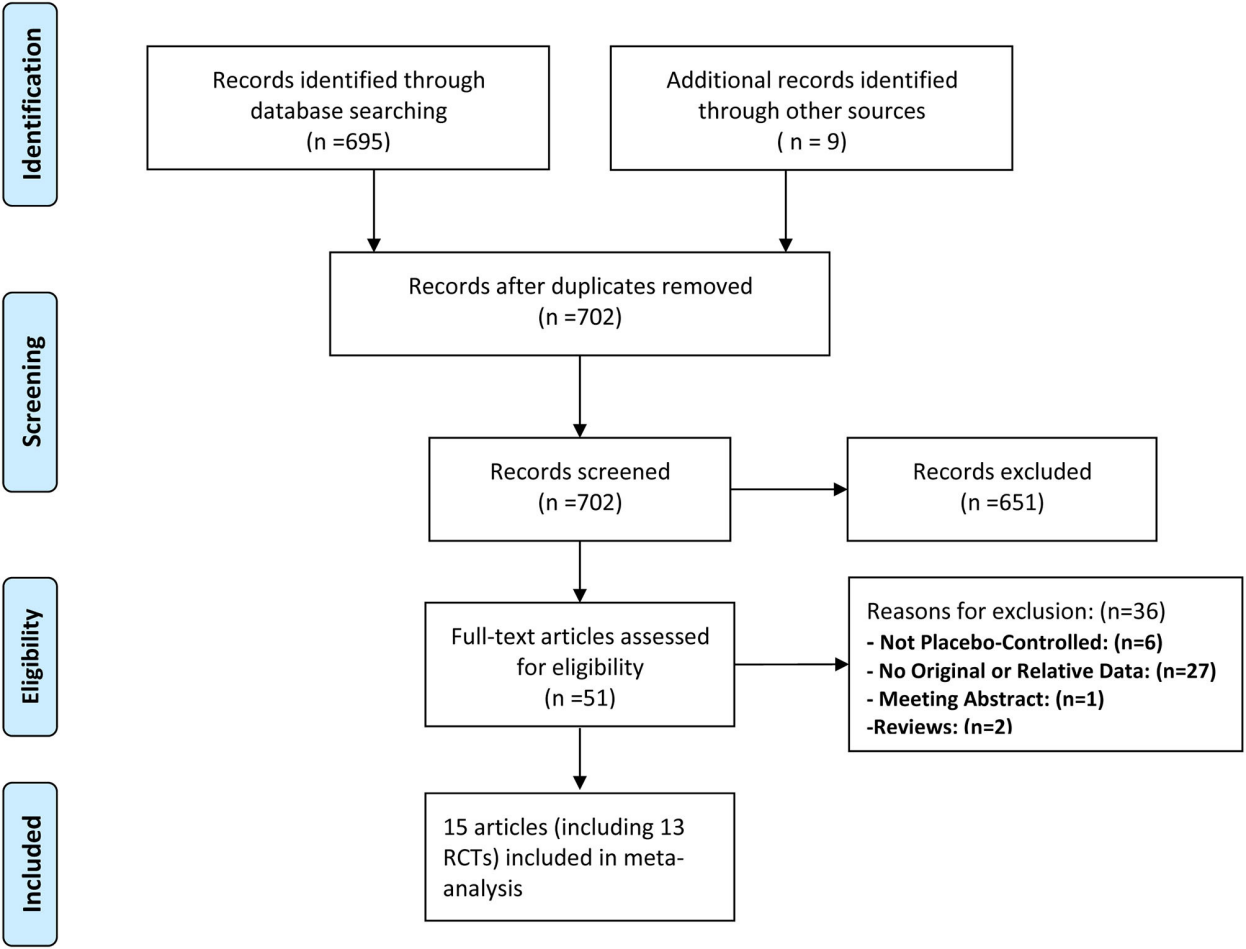
Preferred Reporting Items for Systematic Reviews and Meta-analysis (PRISMA) flow diagram of study selection.

Two investigators independently conducted title and abstract selection and full-text review. The PRISMA flowchart about the selection process are displayed in Figure 1. Two reviewers extracted data from all included studies, such as author (year), sample size, age, cancer status, follow-up time, duration of treatment, and interested outcomes. Any disagreements were resolved by a third reviewer.

### Outcomes

The primary outcomes of the present meta-analysis were risk ratio (*RR*) for any-grade diarrhea and constipation of patients receiving any types of novel ARAT agents compared with control group. The secondary outcomes included: (a) combined incidence of any-grade and grade 3 or greater diarrhea or constipation in the total ARAT group, ARAT subgroups (Abi, Enz, Apa, and Dar), and control groups; (b) RR for grade 3 or greater diarrhea and constipation of patients receiving any types of novel ARAT agents. Any-grade adverse events are defined as events from grade 1 to higher grades.

### Risk of Bias Assessment

Risk of bias (RoB) was independently determined by two investigators using the Cochrane Collaboration's tool (version 2.0) (21). To assess the RoB, 5 domains were considered: bias arising from the randomization process, bias due to deviations from intended interventions, bias due to missing outcome data, bias in measurement of the outcome, and bias in selection of the reported result. If the study is judged to be at low RoB for all domains for a specific result, the overall RoB would be low. If at least one domain was judged to be at high RoB or multiple domains were judged to have some concerns for a specific result, the overall RoB would be high. Any disagreements were resolved by a third investigator.

### Statistical Analysis

Risk ratio with 95% *CI*s were estimated for diarrhea and constipation using pairwise and network meta-analysis. The analysis was performed in two steps: first, pairwise meta-analysis was performed to assess a particular outcome. The Mantel–Haenszel (M–H) random-effects model was applied to pooled RRs for any-grade and grade 3 or greater diarrhea or constipation, given the expected heterogeneity within part of the evaluated trials. Heterogeneity across studies was formally tested for using chi-square (*p* < 0.05) and the *I*^2^ statistic. Subgroup analysis was based on the different types of ARAT agents that patients received in intervention groups. Publication bias was evaluated utilizing the funnel plots and the Peters test of funnel plot symmetry (22). Considering the different disease stages of our included studies, a subgroup analysis was performed for patients with mHSPC and mCRPC/nmCRPC. A *p* < 0.05 suggested significantly statistical risk of publication bias.

Second, Bayesian analyses were conducted for network meta-analysis with random-effects for any-grade diarrhea or constipation (23). To assess whether there was inconsistency between direct and indirect comparisons, we compared the random effects variances of consistency and inconsistency model for all the outcomes, which indicated similar random effects SD between the two models. Therefore, the results of consistency models were reported. Convergence is assessed using the Brooks-Gelman-Rubin method. This method compares within-chain and between-chain variance to calculate the potential scale reduction factor (PSRF). A PSRF close to 1 indicates that approximate convergence has been reached (24). There were 4 chains, initial values scaling was 2.5, tuning iterations were 20,000, simulation iterations were 50,000, and thinning interval was 10. For each iteration, the ranking of Abi, Enz, Apa, Dar, and placebo was determined using the RRs from that iteration. Surface under the cumulative ranking curve (SUCRA) was calculated from these rankings by summing the cumulative probabilities of all the ranks divided by the number of ranks minus 1 (25). This statistic has no known distribution and is a means of summarizing treatment rankings.

The significant level was *p* < 0.05 for statistical tests. All statistical analyses were performed and forest plots generated using the “gemtc,” “rjags,” “meta,” and “metafor” packages from R 3.6.2 (R project) and Review Manager v5.2 software.

## Results

### Study Selection and Network Geometry

A total of 702 unique records were screened for eligibility, of which 651 were excluded based on the evaluation of titles and abstracts. Full-text screening was assessed for 51 articles, eventually, 15 unique studies (such as 13 RCTs) fulfilled inclusion criteria (Figure 1).

**Figure 2:**
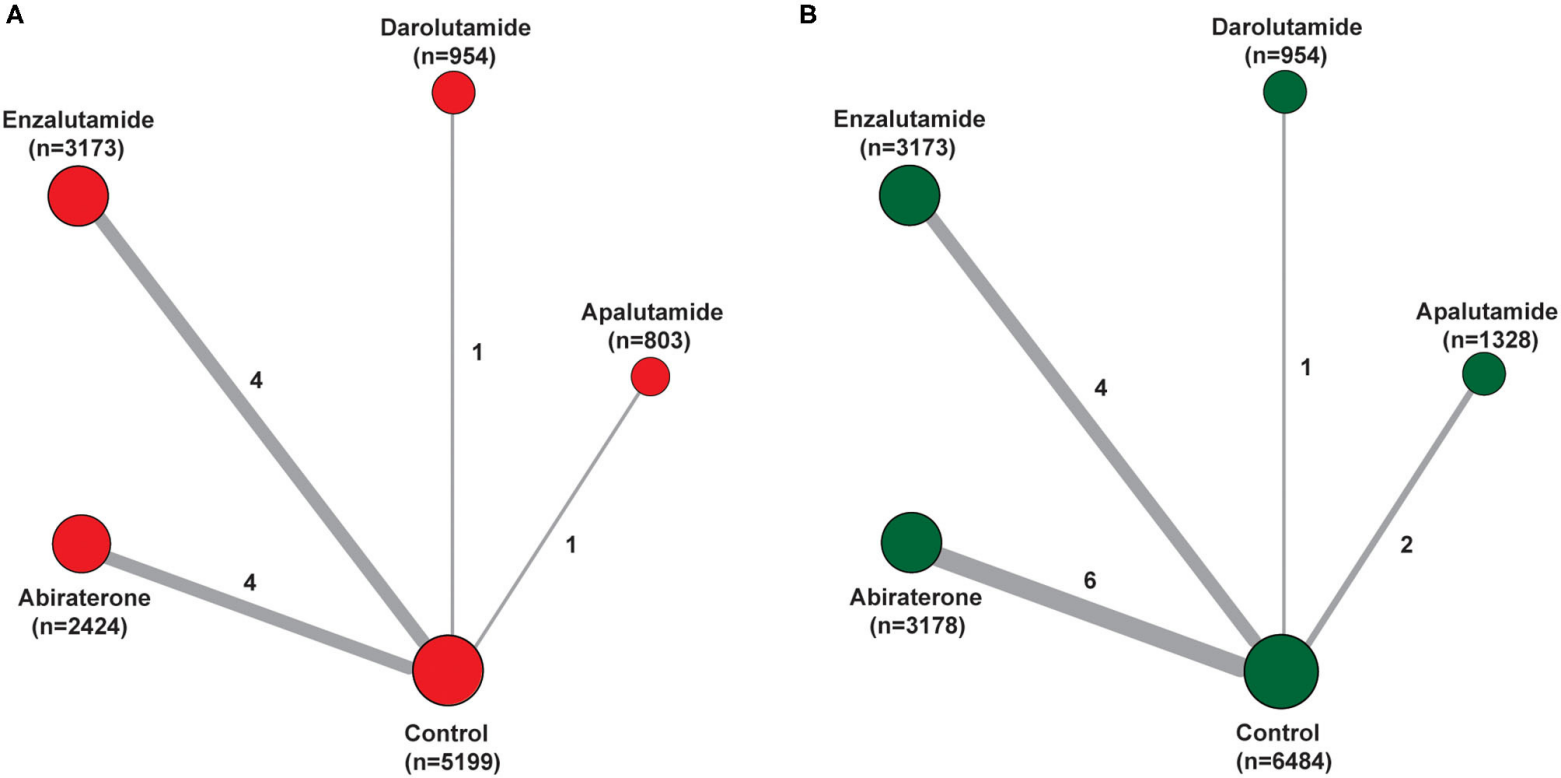
Network of the comparisons for the Bayesian network meta-analysis. **(A)** Diarrhea; **(B)** constipation. The size of the nodes is proportional to the number of patients (in parentheses) randomized to receive the treatment. The width of the lines is proportional to the number of trials (beside the line) comparing the connected treatments.

In total, 13 trials comparing five treatments were assessed, including placebo, Abi, Enz, Apa, and Dar (Table 1). The well-connected network structure for both diarrhea and constipation are displayed in Figures 2A,B. The width of the lines represents the number of trials comparing each pair of treatments. The size of the circle represents the sample size in each arm. More details of numbers of trials and sample size of each treatment are presented in Figure 2.

**Table 1:** Baseline characteristics of included studies.

**References**	**Trial**	**Phase**	**Intervention**	**Patients, No**.			**Cancer status**	**Age, Median/Mean (Range/SD)**		**Duration of treatment, Median, mo**	
				**Total**	**Experimental**	**Control**		**Experimental**	**Control**	**Experimental**	**Control**
Smith et al. (26)	SPARTAN	3	Apalutamide	1,201	803	398	nmCRPC	74 (48–94)	74 (52–97)	60.9%	29.9%
Fizazi et al. (27); Fizazi et al. (28)	ARAMIS	3	Darolutamide	1,508	954	554	nmCRPC	74 (48–95)	74 (50–92)	14.8	11.6
Sternberg et al. (29); Hussain et al. (30)	PROSPER	3	Enzalutamide	1,395	930	465	nmCRPC	74 (50–95)	73 (53–92)	18.4	11.1
Beer et al. (31)	PREVAIL	3	Enzalutamide	1,715	871	844	mCRPC	72 (43–93)	71 (42–93)	16.6	4.6
Chi et al. (8)	TITAN	3	Apalutamide	1,052	525	527	mHSPC	69 (45–94)	68 (43–90)	20.5	18.3
Armstrong et al. (7)	ARCHES	3	Enzalutamide	1,146	572	574	mHSPC	70 (46–92)	70 (42–92)	12.8	11.6
Fizazi et al. (5)	LATITUDE	3	Abiraterone	1,199	597	602	mHSPC	68 (38–89)	67 (33–92)	24	14
James et al. (4)	STAMPEDE	3	Abiraterone	1,908	948	960	mHSPC	67 (42–85)	67 (39–84)	23.7	NR
Ryan et al. (32)	COU–AA−302	3	Abiraterone	1,082	542	540	mCRPC	71 (44–95)	70 (44–90)	NR	NR
Scher et al. (33)	AFFIRM	3	Enzalutamide	1,199	800	399	CRPC	69 (41–92)	69 (49–89)	8.3	3.0
de Bono et al. (34)	COU–AA−301	3	Abiraterone	1,185	791	394	mCRPC	69 (42–95)	69 (39–90)	8	4
Ye et al. (35)	–	3	Abiraterone	313	157	156	mCRPC	69.7 (8.72)^*^	70.8 (8.64)^*^	3.8	3.4
Sun et al. (36)	–	3	Abiraterone	214	143	71	mCRPC	68.2 (8.30)^*^	67.7 (7.75)^*^	8.1	4.2

*NO, Number; nmCRPC, nonmetastatic castration–resistant prostate cance; mCRPC, metastatic castration–resistant prostate cancer; mHSPC, metastatic hormone–sensitive prostate cancer; CRPC, castration–resistant prostate cancer; NR, Not Reported*.

* *Mean (SD)*.

### Characteristics of Included Trials

Tables 1, 2 presented the characteristics of included studies. All 13 included trials were phase 3, double-blind, placebo-controlled, randomized studies published between 2011 and 2020, involving a total of 15,117 patients (6,484 in control group, 3,178 in Abi group, 3,173 in Enz group, 1,328 in Apa group, and 954 in Dar group). Median age of participants in ARAT and control group were both ranged from 67 to 74. Ten trials reported the related data of diarrhea, and all 13 trials displayed the data of constipation. There were 4, 3, 5, and 1 trials included patients diagnosed with mHSPC, nmCRPC, mCRPC, and CRPC, respectively. The median (range) duration of treatment was 16.6 (8.0–24.0) month in the ARAT group and 11.6 (3.0–18.3) month in the control group. For diarrhea, 6 trials were assessed with low overall RoB (60.0%) and 4 were judged as some concerns (40.0%). For constipation, the overall RoB was low in six trials (46.2%) and the remaining seven trials raised some concerns (53.8%) (Table 2). As for publication bias, statistical analysis of Peters test indicated no evidence of publication bias both in diarrhea and constipation (*p* were 0.82 and 0.63, respectively) (Supplementary Figures 1, 2).

**Table 2:** Risk of bias within trials.

**Trial**	**R**	**D**	**Mi**	**Me**	**S**	**O**
**Diarrhea**						
SPARTAN	Low	Low	Low	Low	Low	Low
ARAMIS	Low	Low	Low	Low	Low	Low
PROSPER	Low	Low	Low	Some concerns	Low	Some concerns
PREVAIL	Low	Low	Low	Low	Low	Low
ARCHES	Low	Low	Low	Low	Low	Low
STAMPEDE	Low	Low	Some concerns	Low	Low	Some concerns
COU-AA-302	Low	Low	Low	Some concerns	Low	Some concerns
AFFIRM	Low	Low	Low	Low	Low	Low
COU-AA-301	Low	Low	Low	Low	Low	Low
Sun et al. (36)	Low	Low	Low	Some concerns	Low	Some concerns
**Constipation**						
SPARTAN	Low	Low	Low	Low	Low	Low
ARAMIS	Low	Low	Low	Low	Low	Low
PROSPER	Low	Low	Low	Some concerns	Low	Some concerns
PREVAIL	Low	Low	Low	Low	Low	Low
TITAN	Low	Low	Low	Some concerns	Low	Some concerns
ARCHES	Low	Low	Low	Low	Low	Low
LATITUDE	Low	Low	Low	Some concerns	Low	Some concerns
STAMPEDE	Low	Low	Some concerns	Low	Low	Some concerns
COU-AA-302	Low	Low	Low	Some concerns	Low	Some concerns
AFFIRM	Low	Low	Low	Low	Low	Low
COU-AA-301	Low	Low	Low	Low	Low	Low
Ye et al. (35)	Low	Low	Low	Some concerns	Low	Some concerns
Sun et al. (36)	Low	Low	Low	Some concerns	Low	Some concerns

*Risk of bias legend: R, Bias arising from the randomisation process; D, Bias due to deviations from intended interventions; Mi, Bias due to missing outcome data; Me, Bias in measurement of the outcome; S, Bias in selection of the reported result; O, Overall risk of bias*.

### Safety Assessment

#### Incidence of Diarrhea and Constipation

In ARAT group, the reported any-grade diarrhea was 1,309 (17.8%) and that of grade 3 or greater diarrhea was 45 (0.5%). In the control group, the reported any-grade diarrhea was 756 (14.5%) and that of grade 3 or greater diarrhea was 24 (0.3%). The reported incidence of constipation in the ARAT group was 1,463 (16.9%) and that grade 3 or greater constipation was 28 (0.2%). The reported incidence of constipation in the control group was 991 (15.3%) and that grade 3 or greater constipation was 24 (0.2%) (Table 3). As for the reported incidence of any-grade diarrhea in groups of individual ARAT agents, Abi had the highest rate at 25.4% (95% *CI* [23.7–27.2%]), followed by Apa at 20.3% (95% *CI* [17.7%, 23.2%]), followed by Enz at 14.5% (95% *CI* [13.3%, 15.7%]), followed by Dar at 7.4% (95% *CI* [5.9%, 9.3%]) (Supplementary Table 1). Similarly, the highest rate of any-grade diarrhea in individual control groups was also Abi, followed by Apa, Enz, and Dar (Supplementary Table 1). As for the rate of any-grade constipation in groups of individual ARAT agents, Abi was associated with the highest rate at 22.3% (95% *CI* [20.8%, 23.7%]), followed by Enz at 17.5% (95% *CI* [16.2%, 18.9%]), Apa at 10.1% (95% *CI* [8.8%, 11.8%]), and Dar at 6.9% (95% *CI* [5.5%, 8.7%]) (Supplementary Table 1). The sequence of incidence of any-grade constipation in individual control groups was also similar with the sequence of ARAT groups (Supplementary Table 1).

**Table 3:** Pooled analysis of ARAT use with diarrhea and constipation risk.

**Adverse event**	**Experimental groups**			**Control groups**			**Pool estimate**			
	**Patients, No**.	**Adverse events, No**.	**Incidence (%)**	**Patients, No**.	**Adverse events, No**.	**Incidence (%)**	**Studies, No**.	**RR (95% CI)**	***p* value**	**I^**2**^**
Diarrhea										
All grades	7,354	1,309	17.80	5,199	756	14.54	10	1.30 (1.16, 1.44)	<0.001	32%
Grade ≥3	7,354	45	0.61	5,199	23	0.44	10	1.24 (0.73, 2.09)	0.43	0%
Constipation										
All grades	8,633	1,463	16.94	6,484	991	15.28	13	1.08 (0.95, 1.22)	0.25	59%
Grade ≥3	8,458	28	0.33	6,328	24	0.38	12	0.84 (0.49, 1.46)	0.54	0%

*No, Number; RR, Risk Ratio*.

#### Risk of Diarrhea

**Figure 3:**
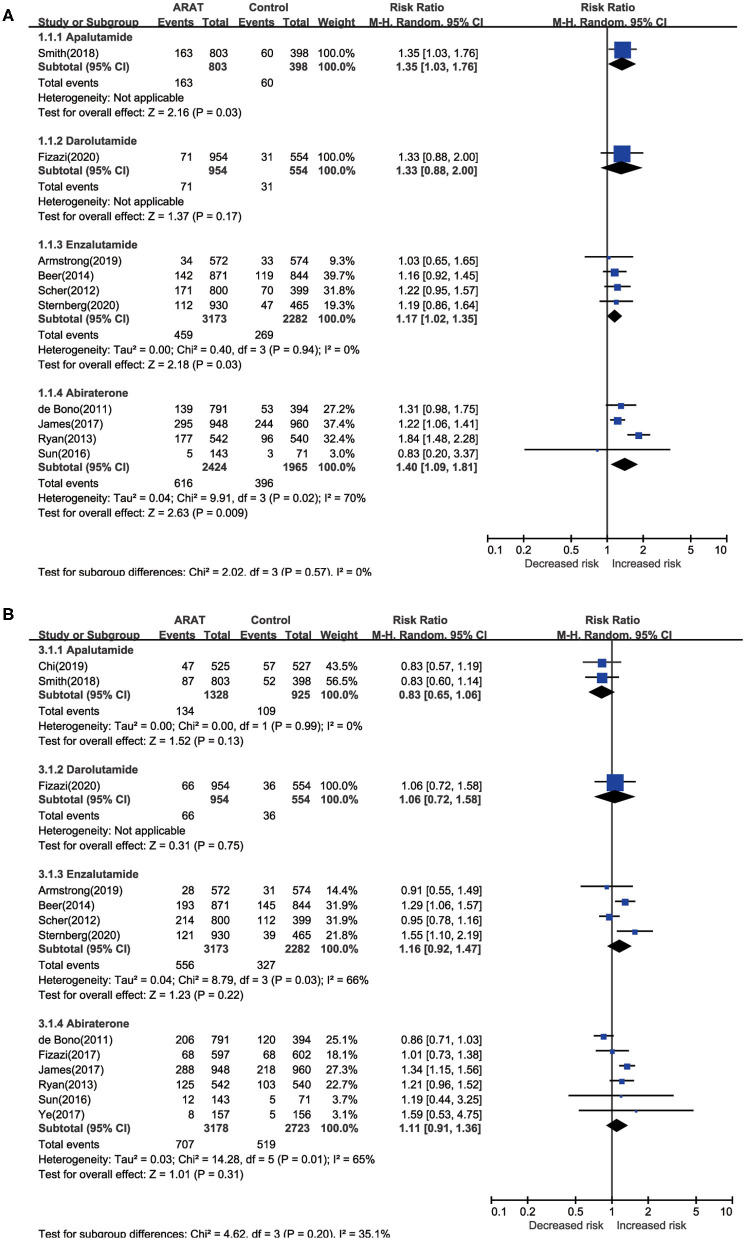
Pairwise meta-analysis for risk of diarrhea and constipation among included ctudies. **(A)** Diarrhea; **(B)** constipation.

Pairwise meta-analysis indicated significantly increased risk of any-grade diarrhea for patients treated with novel ARAT agents compared with placebo (*RR* = 1.30, 95% *CI* [1.16, 1.44], *I*^2^ = 32%) (Table 3). As for subgroup analysis, all the four novel ARAT agents had the potential to increase the risk of any-grade diarrhea, moreover, treated with Abi, Enz, and Apa could significantly increase the risk of any-grade diarrhea compared with placebo group (Abi vs. placebo: *RR* = 1.40, 95% *CI* [1.09, 1.81]; Enz vs. placebo: *RR* = 1.17, 95% *CI* [1.02, 1.35]; Apa vs. placebo: *RR* = 1.35, 95% *CI* [1.03, 1.76]; and Dar vs. placebo: *RR* = 1.33, 95% *CI* [0.88, 2.00]) (Figure 3A). As for the grade 3 or greater diarrhea, there was no significant difference between ARTA and placebo group (ARAT vs. placebo: *RR* = 1.24, 95% *CI* [0.73, 2.09], *I*^2^ = 0%) (Table 3). Subgroup analysis indicated consistent results for risk of any-grade diarrhea between patients with mHSPC and mCRPC/nmCRPC (*p* = 0.321) (Supplementary Table 2).

**Figure 4:**
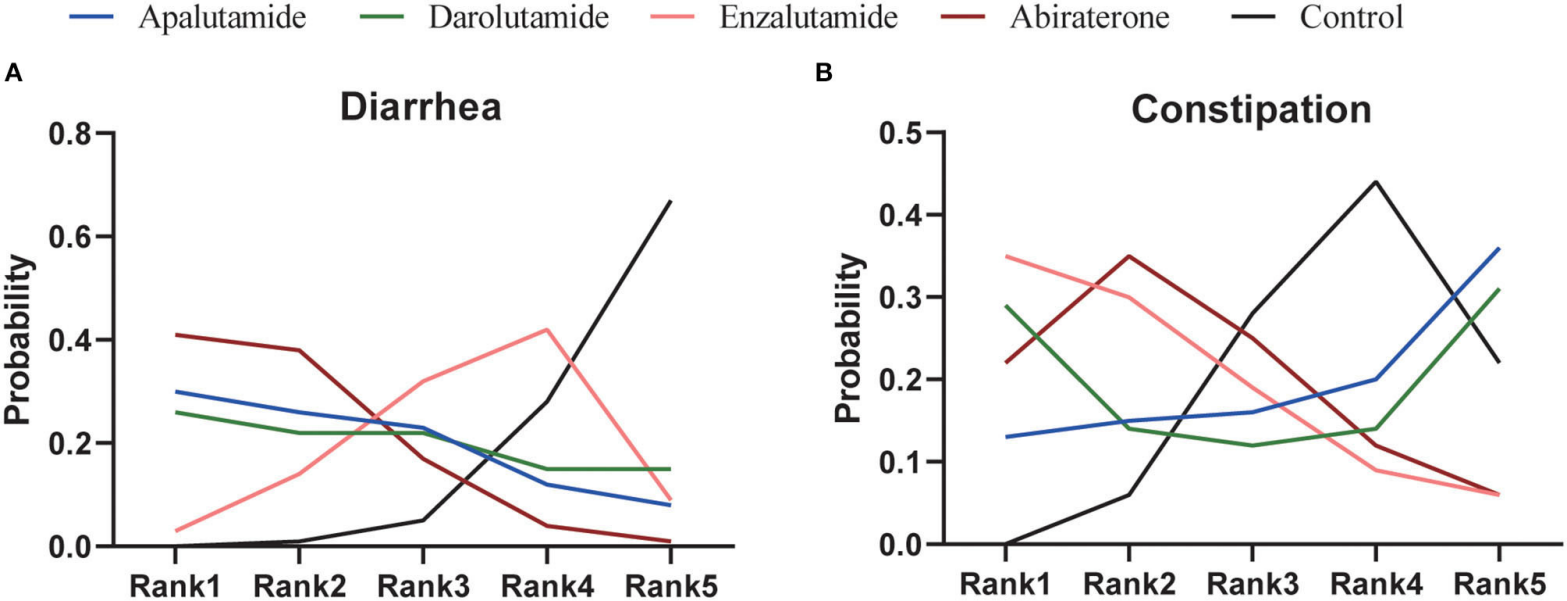
Ranking of treatments in terms of diarrhea **(A)** and constipation **(B)**.

As for network meta-analysis, Abi could significantly increase the risk of any-grade diarrhea compared with placebo (*RR* = 1.55, 95% *CI* [1.08, 2.13]) (Table 4). Although there were no significant differences, Enz, Apa, and Dar could also show the potential for increased risk of any-grade diarrhea (Table 4). Furthermore, risk of any-grade diarrhea among the four novel ARAT agents did not show significant difference (Table 4). Based on Bayesian modeling, Abi showed the highest probability to rank first, Apa and Dar displayed similar probability to rank first, and Enz showed lowest probability to rank first in terms of increasing risk of any-grade diarrhea (Figure 4). Last, we ranked the probability that each ARAT agents (Abi, Enz, Apa, and Dar) resulted in higher risk of diarrhea using the SUCRA. The SUCRA for Abi, Enz, Apa, Dar, and placebo were 76, 40, 64, 62, and 8% for risk of any-grade diarrhea, respectively.

**Table 4:** Network meta-analysis for RR of diarrhea (below diagonal) and constipation (above diagonal).

**Abiraterone**	1.15 (0.66, 1.99)	1.08 (0.51, 2.07)	0.96 (0.63, 1.52)	1.15 (0.87, 1.54)
1.08 (0.51, 2.15)	**Apalutamide**	0.93 (0.40, 2.15)	0.83 (0.47, 1.50)	1.00 (0.62, 1.60)
1.13 (0.53, 2.46)	1.06 (0.42, 2.72)	**Darolutamide**	0.90 (0.42, 1.95)	1.07 (0.54, 2.16)
1.30 (0.79, 2.06)	1.20 (0.60, 2.48)	1.13 (0.53, 2.42)	**Enzalutamide**	1.20 (0.86, 1.66)
**1.55 (1.08, 2.13)**	1.43 (0.77, 2.69)	1.36 (0.67, 2.67)	1.19 (0.85, 2.64)	**Control**

*Bold values indicate statistically significant*.

#### Risk of Constipation

For the risk of constipation, the pairwise meta-analysis did not show significant difference both in any-grade and grade 3 or greater between ARAT and control group (Table 3). As for subgroup analysis, all the four agents could not significantly influence the risk of any-grade constipation compared with placebo (Figure 3B). Additionally, subgroup analysis suggested consistent results for risk of any-grade constipation between patients with mHSPC and mCRPC/nmCRPC (*p* = 0.992) (Supplementary Table 2).

Network meta-analysis showed consistent results with pairwise meta-analysis between ARAT and control group (Table 4). Similarly, risk of any-grade constipation among the four novel ARAT agents did not show significant difference (Table 4). Bayesian modeling showed that Enz had the highest probability to increase the risk of any-grade constipation (Figure 4). As for SUCRA, Abi, Enz, Apa, Dar, and placebo were 64, 77, 15, 56, and 38% for risk of any-grade constipation, respectively.

## Discussion

Across 13 RCTs, such as patients with CaP treated with novel ARAT agents, we found that the use of ARAT agents was associated with the risk of diarrhea. The use of ARAT agents was associated with 1.30 times higher risk of diarrhea. As for individual ARAT agents, Abi, Enz, Apa, and Dar were associated with 1.40, 1.17, 1.35, and 1.33 times higher risk of diarrhea, respectively. There were no significant differences for risk of constipation between ARAT and control groups. Based on Bayesian modeling, Abi might be associated with highest risk of diarrhea, among the four ARAT agents.

The sequence of incidence for diarrhea and constipation among groups of individual ARAT agents might be caused by both the heterogeneity of participants and the effect of agents, which could be evidenced by similar sequence of incidence in individual control groups and different *RR* of each ARAT agents for risk of diarrhea. The present study highlighted several insights. First, we evidenced that novel ARAT agents could significantly increase the risk of diarrhea for patients with CaP based on data from phase 3, placebo-controlled, double-blind, and randomized trials. There were potential links between CaP and treatment and increased inflammatory levels from GI dysbiosis (37). As mentioned, the components of GI microbiota could be affected by serum androgen levels and castration (14–16), and both Enz and Abi could alter the components of GI microbiota (17). Recently, Pernigoni et al. indicated that the GI microbiota could also provide an alternative source of androgen in patients and mice with CRPC (38). Therefore, our results indirectly evidenced the hypothesis that we mentioned previously.

Second, based on our results, we might highlight two potential interventions for diarrhea caused by novel ARAT gents, especially patients treated with Abi, which were probiotics and exercise. Many elderlies are less fit physiologically to withstand the effect of diarrhea on fluid balance and nutritional balance (39), therefore, it is necessary to intervene in the treatment-related diarrhea for the elderly. Previous studies have shown that probiotics supplements could modify the GI side effects induced by radiotherapy, chemotherapy, and immunotherapy, as the three treatments modality could induce GI dysbiosis and subsequently cause diarrhea, mucositis, and so on (40). Even though, probiotics supplements could enhance the action of chemotherapy drugs. Therefore, considering the high incidence and significant increased risk of diarrhea in patients receiving treatment of novel ARAT agents, it is necessary to explore the role of probiotics supplements in toxicity modification and treatment action of novel ARAT agents. Perhaps unsurprisingly, emerging evidence has suggested that exercise might exert a positive effect on the components of GI microbiota (41). The results of a meta-analysis included 14 RCTs supported a strong recommendation for supervised exercise therapy for improving disease-specific quality of life in patients receiving ADT (42). Excitedly, an ongoing single-blinded, two-armed, RCT was designed to explore the influence of a 3-month exercise program (3 days/week) for gut health in men receiving ADT (37).

Our study has some limitations. First, clinical consequences of diarrhea and constipation on therapy, and the use of potential interventions were not reported in our included studies. Therefore, the two potential interventions that we mentioned needs further investigations. Second, we could not conduct age-stratified or other subgroup analysis for risk of diarrhea or constipation, because the cut-off levels were different across trials and the included studies were not focused on reporting risk factors for diarrhea and constipation related to age or other valuables. Third, the duration of treatment was different across trials. The relatively short therapy duration for novel ARAT agents may bias against their long-term effectiveness estimation. Interestingly, meta-regression analysis regarding the duration of hormone therapy both for risk of diarrhea and constipation indicated that the duration of hormone therapy might not affect the stability of our present results (*p* = 0.963 and 0.062 for risk of diarrhea and constipation). Fourth, there were no time-based data to calculate the diarrhea and constipation person-year incidence rates. Fifth, the present study only included 2 and 1 trials focused on Apa and Dar, therefore, it might need further update. Sixth, the present results are from network meta-analysis, therefore, prospective clinical trials regarding this issue are suggested in the future.

## Conclusion

The present study indicates that the use of novel ARAT agents is associated with a significantly higher risk of diarrhea. Across the four agents, Abi may relate to the highest risk of diarrhea and Enz may relate to the lowest risk of diarrhea among patients with mHSPC and CRPC. Considering the high incidence and significantly increased risk of diarrhea in patients receiving novel ARAT agents, it is necessary to develop potential interventions regarding the novel ARAT agent-related diarrhea.

## Data Availability Statement

The original contributions presented in the study are included in the article/Supplementary Material, further inquiries can be directed to the corresponding author/s.

## Author Contributions

LY and SQ were responsible for the conception, design of the study, and obtained public funding. XX, HX, and SW did the analysis and interpreted the analysis. XL, XY, and KJ were responsible for the acquisition of data. XX, HX, SB, and HL wrote the first draft of the manuscript. KJ, SB, and LY interpreted the data and wrote the final version. All authors critically revised the article for important intellectual content and approved the final version.

## Funding

This work was supported by the National Key Research and Development Program of China (Grant No. 2017YFC0908003), the National Natural Science Foundation of China (Grant Nos. 81974099, 81974098), the Post-doctoral Science Research Foundation of Sichuan University (2020SCU12041), the National Clinical Research Center for Geriatrics, West China Hospital, Sichuan University (Z2018C01), the Technology Innovation Research and Development Project, Chengdu Science and Technology Bureau (2019-YF05-00296-SN), the Clinical Research Incubation Project of West China Hospital of Sichuan University (2020HXFH033), and the China Society of Clinical Oncology—Pilot Oncology Research Fund (Y-2019AZMS-0523).

## Conflict of Interest

The authors declare that the research was conducted in the absence of any commercial or financial relationships that could be construed as a potential conflict of interest.

## Publisher's Note

All claims expressed in this article are solely those of the authors and do not necessarily represent those of their affiliated organizations, or those of the publisher, the editors and the reviewers. Any product that may be evaluated in this article, or claim that may be made by its manufacturer, is not guaranteed or endorsed by the publisher.

## Supplementary Material

Supplementary Figure 1 Funnel plot for the included studies of diarrhea.

Supplementary Figure 2 Funnel plot for the included studies of constipation.

Supplementary Table 1 Outcomes of reported diarrhea and constipation adverse events (AEs) in combined studies.

Supplementary Table 2 Subgroup analysis of ARAT use with diarrhea and constipation risk.
