# Supplementary figures and images for: Association of Novel Androgen Receptor Axis-Targeted Therapies With Diarrhea in Patients With Prostate Cancer: A Bayesian Network Analysis
Source: Front Med (Lausanne). 2022 Jan 24;8:800823. doi: 10.3389/fmed.2021.800823 (PMC8818787; doi:10.3389/fmed.2021.800823)

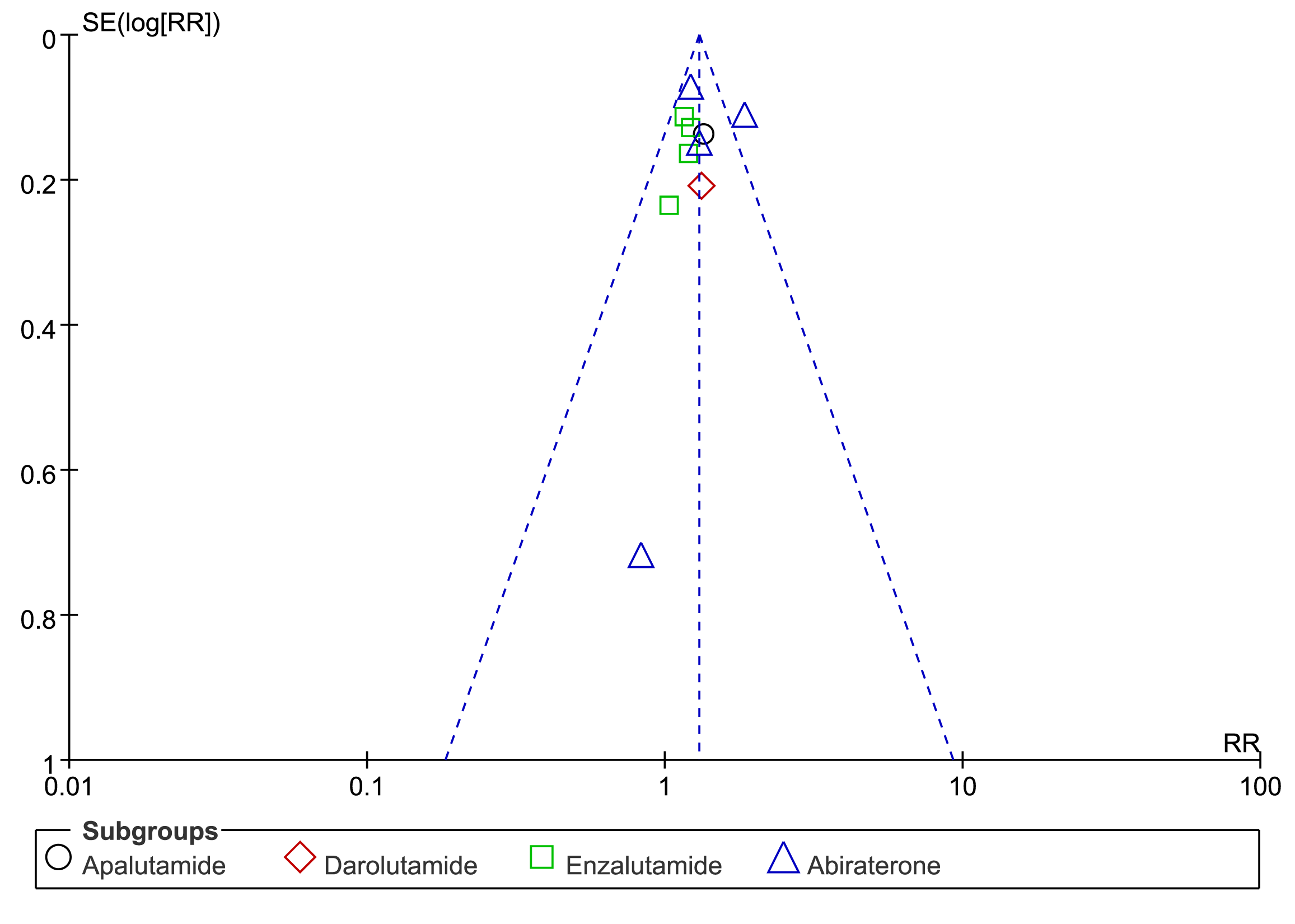

Supplement: Supplementary Figure 1 — Funnel plot for the included studies of diarrhea. [file Image_1.TIF]

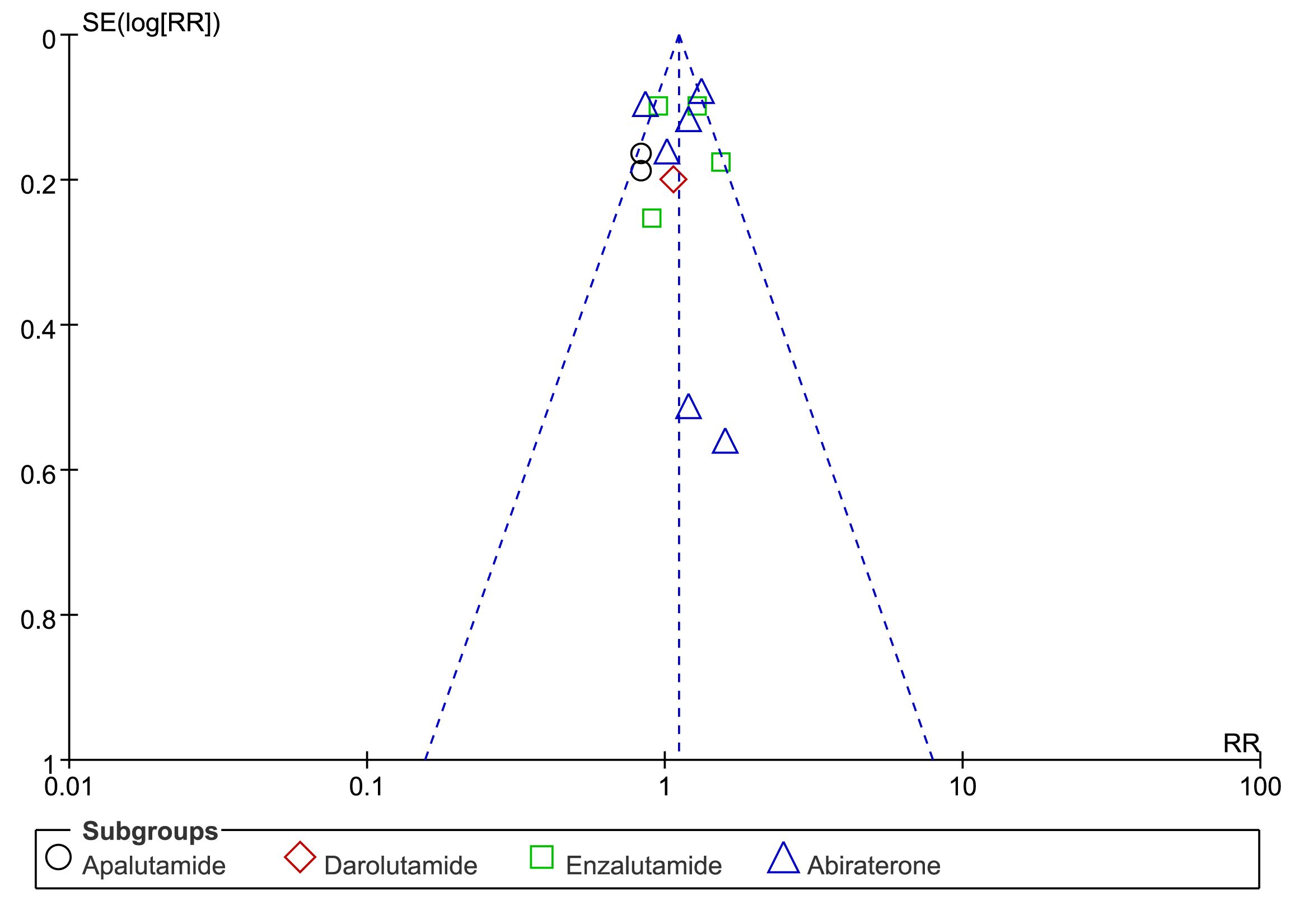

Supplement: Supplementary Figure 2 — Funnel plot for the included studies of constipation. [file Image_2.TIF]
