# Supplementary material for: Association of Novel Androgen Receptor Axis-Targeted Therapies With Diarrhea in Patients With Prostate Cancer: A Bayesian Network Analysis
Source: Front Med (Lausanne). 2022 Jan 24;8:800823. doi: 10.3389/fmed.2021.800823 (PMC8818787; doi:10.3389/fmed.2021.800823)
Supplement: Supplementary Table 1 — Outcomes of reported diarrhea and constipation adverse events (AEs) in combined studies. [file Table_1.docx]

|  | **Diarrhea (ARAT groups), Events/Total (****%, [95%CI])** | | **Diarrhea (Control groups), Events/Total (%, [95%CI])** | | **Constipation (ARAT groups), Events/Total (%, [95%CI])** | | **Constipation (Control groups), Events/Total (%, [95%CI])** | |
| --- | --- | --- | --- | --- | --- | --- | --- | --- |
|  | **Any-grade** | **grade ≧3** | **Any-grade** | **grade ≧3** | **Any-grade** | **grade ≧3** | **Any-grade** | **grade ≧3** |
| **Abi** | 616/2424 (25.4[23.7, 27.2]) | 20/2424 (0.7[0.4, 1.1]) | 396/1965 (20.2[18.4, 22.0]) | 13/1965 (0.4[0.1, 0.8]) | 707/3178 (22.3[20.8, 23.7]) | 16/3021 (0.4[0.2, 0.7]) | 519/2723 (19.1[17.6, 20.6]) | 16/2567 (0.4[0.2, 0.7]) |
| **Enz** | 459/3173 (14.5[13.3, 15.7)] | 16/3173 (0.4[0.2, 0.7]) | 269/2282 (11.8[10.5, 13.2]) | 7/2282  (0.3[0.1, 0.6]) | 556/3173 (17.5[16.2, 18.9]) | 12/3173 (0.3[0.1, 0.6]) | 327/2282 (14.3[13.0, 15.8]) | 8/2282 (0.3[0.1, 0.6]) |
| **Apa** | 163/803  (20.3[17.7, 23.2]) | 8/803  (1.0[0.4, 1.8]) | 60/398  (15.1[11.2, 18.9]) | 3/398  (0.5[0.0, 1.5]) | 134/1328 (10.1[8.8, 11.8]) | 0/1328  (0.0[0.0, 0.1]) | 109/925 (11.8[9.9, 14.3]) | 0/925  (0.0[0.0, 0.2]) |
| **Dar** | 71/954  (7.4[5.9, 9.3]) | 1/954  (0.1[0.0, 0.4]) | 31/554  (5.6[4.0, 7.9]) | 1/554  (0.2[0.0, 0.8]) | 66/954  (6.9[5.5, 8.7]) | 0/954  (0.0[0.0, 0.2]) | 36/554  (6.5[4.7, 8.9]) | 0/554  (0.0[0.0, 0.3]) |
| **Combined** | 1309/7354 (17.8[16.9, 18.7]) | 45/7354 (0.5[0.3, 0.7]) | 756/5199 (14.5[13.6, 15.5]) | 24/5199 (0.3[0.2, 0.5]) | 1463/8633 (16.9[16.2, 17.8]) | 28/8476 (0.2[0.1, 0.3]) | 991/6484 (15.3[14.4, 16.2]) | 24/6328 (0.2[0.1, 0.4]) |

Supplemental Table 1. Outcomes of Reported Diarrhea and Constipation Adverse Events (AEs) in Combined Studies

Abi=abiraterone; Enz=Enzalutamide; Apa=Apalutamide; Dar=Darolutamide.
