# Supplementary material for: Association of Novel Androgen Receptor Axis-Targeted Therapies With Diarrhea in Patients With Prostate Cancer: A Bayesian Network Analysis
Source: Front Med (Lausanne). 2022 Jan 24;8:800823. doi: 10.3389/fmed.2021.800823 (PMC8818787; doi:10.3389/fmed.2021.800823)
Supplement: Supplementary Table 2 — Subgroup analysis of ARAT use with diarrhea and constipation risk. [file Table_2.docx]

| Adverse event | Experimental Groups | | | Control groups | | | Pool estimate | | |
| --- | --- | --- | --- | --- | --- | --- | --- | --- | --- |
|  | Patients, No. | adverse events, No. | Incidence(%) | Patients, No. | adverse events, No. | Incidence(%) | Studies, No. | RR (95% CI) | p value |
| Diarrhea |  |  |  |  |  |  |  |  | 0.321 |
| mHSPC | 1520 | 329 | 21.64 | 1534 | 277 | 18.06 | 2 | 1.21(1.05, 1.38) |  |
| mCRPC/nmCRPC | 5834 | 980 | 16.80 | 3665 | 479 | 13.07 | 8 | 1.33(1.16, 1.53) |  |
| Constipation |  |  |  |  |  |  |  |  | 0.992 |
| mHSPC | 2642 | 431 | 16.31 | 2663 | 374 | 14.04 | 4 | 1.06(0.83, 1.35) |  |
| mCRPC/nmCRPC | 5991 | 1032 | 17.23 | 3721 | 617 | 16.58 | 9 | 1.05(0.92, 1.21) |  |

Supplemental Table 2. Subgroup Analysis of ARAT Use With Diarrhea and Constipation Risk.

No=Number; RR=Risk Ratio; mHSPC=metastatic hormone-sensitive prostate cancer; mCRPC=metastatic castration-resistant prostate cancer; nmCRPC=nonmetastatic castration-resistant prostate cance.
